# The permanent magnet hypothesis: an intuitive approach to designing non-circular magnet arrays with high field homogeneity

**DOI:** 10.1038/s41598-023-29533-9

**Published:** 2023-02-16

**Authors:** Sumit Tewari, Andrew Webb

**Affiliations:** Center for High Field MRI, Radiology, Leiden University Medical Center, Leiden, The Netherlands

**Keywords:** Applied physics, Techniques and instrumentation, Medical research, Physics, Medical imaging, Magnetic resonance imaging

## Abstract

Does the Halbach magnetization rotation rule that is used for designing circular magnet arrays for achieving the best homogeneity hold also for an elliptical or other non-circular cross-section? In this article, it is shown that a new numerically optimized magnetization rotation rule can provide more than three orders of magnitude improvement in field homogeneity as compared to a Halbach configuration for elliptical systems. Further it is demonstrated that such optimized magnetization rules can be easily derived in an intuitive way by studying virtual permanent magnets of a similar cross-section as the desired magnet array. This is coined as a permanent magnet hypothesis. Finally, it is shown that the applicability of this technique is not limited to circular or elliptical systems but can be applied to any arbitrarily shaped cross-section.

## Introduction

Enhancing the magnetic field on one side of a magnet array while cancelling the field on the other side was first demonstrated by John C. Mallinson in 1973^1^. These single-sided flux generating magnet arrays were designed by changing the orientation of individual permanent magnets in a periodic manner. Klaus Halbach^2^ in 1980 laid down a set of rotation rules which gave rise to different multipole field concentration modes inside a cylindrical magnet array. These rules state that the magnetization along the periphery of the circular cross-section at an azimuth angle $\theta$ should be arranged at a magnetization angle $\alpha$ = (N+1)$\theta$ + $\phi$ , where $\phi$ is a phase factor and N defines the mode. For N = 1, a 2$\theta$ variation is obtained which is a dipole field structure, N = 2 gives a quadrupole field and so on. These single-sided flux generating magnet arrays have found a wide range of applications ranging from magnetic resonance imaging (MRI)^3^, magnetic levitation^4^, focused drug delivery^5^, undulators^6^ to energy harvesting^7^. The rotation rules given by Halbach work best for infinitely long cylindrical arrays with a circular cross-section. When the magnets are arranged in the dipole configuration (N = 1) one obtains a perfectly homogeneous field distribution at the centre of the array. However, practical Halbach cylindrical magnets offinite length suffer from significantly lower field homogeneity.

Much research has concentrated around improving the field homogeneity of a finite-length circular cross-section magnet array consisting of discrete magnet elements. Parameters which have been optimized include the rotation angle of individual magnets, spacing between separate rings of magnets, and the strength and orientation of the magnets themselves^3,8–20^. It is recently demonstrated^21^ that for cylindrical segmented Halbach dipole magnets, the rotation rule itself should be modified for smaller length-to-diameter ratios of Halbach magnets. Instead of an (N+1)$\theta$ variation with N = 1, an additional cos(2$\theta$) term is introduced with a multiplicative coefficient that gets exponentially bigger as the length-to-diameter ratio becomes smaller. An interesting extension, which has not been widely studied, is how to translate all this knowledge to geometries with non-circular cross-sections, for example, an elliptical cross-section. Elliptical cross-section magnet arrays could be useful for MRI applications since the human head or body are intrinsically anatomically elliptical. For this reason, the RF^22–24^ and gradient coils^25,26^ have been designed with elliptical cross-sections. Elliptical resistive magnet designs have also been demonstrated using solenoid type coils^27,28^. Apart from applications in MRI, elliptical magnets are also interesting for magnetic refrigeration systems^29^ and electromagnets in particle accelerators^30^. In these applications, so far only the high magnetic field generation capability of elliptical magnets was used while sacrificing the homogeneity of the field. However, in applications like particle accelerators the homogeneity of the field is also important because it determines the precision with which the particles can be controlled. Other applications where both uniform and strong magnetic fields generated by elliptical cross-section magnets can be used are in NMR spectroscopy, Magnetic resonance force microscopy (MRFM), Atomic clocks and STM, to name a few. Another possible application for non-circular magnet systems could be in the Alpha Magnetic Spectrometer (AMS) systems^31–33^. This is based on the work done by Noble Laureate Prof. Samuel Chao Chung Ting and is used to measure antimatter and dark-matter, and for the precision measurement of the abundance of various isotopes and high energy gamma rays in the universe. These magnetic spectrometer has to be compact and light for it to be installed in the International Space Station (ISS).

There have been only a handful of attempts made in the direction of designing elliptical cross-section permanent magnet arrays. Early in 1983, Gluckstren and Holsinger^34^ studied quadrupoles and dipoles with elliptical cross-sections and used the standard Halbach rotation rules for the magnetization of the individual magnets. Halbach in 1995^35^ discussed magnets with oval inner and outer aperture and studied the effect of ellipticity of these magnets on the field strength inside the magnet. Lee and Jiles^29^ have also discussed the application of such field-enhanced elliptical-shaped Halbach systems in magnetic refrigeration systems. The most recent work on elliptical dipole magnets was by Küstler^11^ in 2010, where the focus was more on the effect of ellipticity on the homogeneity of the field generated. In this work, a finite-size 3D system was studied and the magnetization of the individual magnets was fixed according to the Halbach rotation rules.

In this article, several different new rotation rules for elliptical cross-section magnets are investigated and compared against “Halbach-rotation” in terms of homogeneity. Further an intuitive approach to understanding and designing Halbach systems is introduced. With this approach, it will also be shown that one can derive rotation rules and design magnets for any arbitrarily shaped cross-sections.

## Elliptical Halbach systems

**Figure 1:**
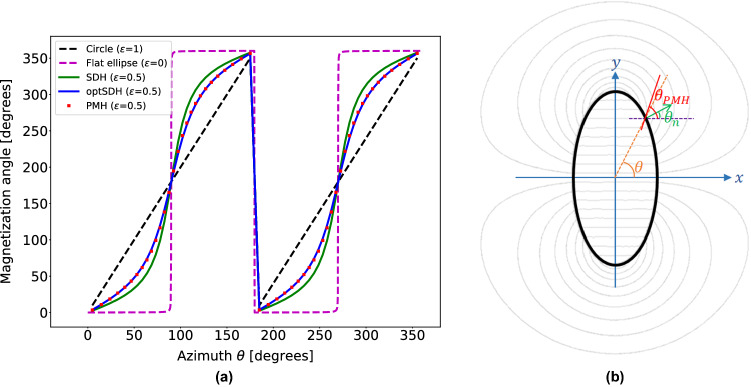
(**a**) A plot of magnetization angle vs azimuth angle ($\theta$) shows that the optimal rotation rules for elliptic magnets are different from the Halbach case for a circular cross-section. The corresponding plots for a magnet with ellipticity 0.5 using a shape-dependent (SDH), optimized shape-dependent (optSDH) and the permanent-magnet hypothesis (PMH) are also shown. (**b**) A pictorial view to explain the three rotation rules. It shows that the Halbach rotation and SDH are geometric relations while PMH on the other hand suggests a connection to a virtual permanent magnet (VPM) as shown here. The magnetization in PMH follows the angle made by the magnetic flux lines emanating from the VPM’s surface i.e., $\theta _{PMH}$.

Does the Halbach rotation rule of 2$\theta$ variation holds also for elliptical cross-section magnets? At the start two extreme cases of zero and unit ellipticity were considered, where ellipticity is given by $\varepsilon =b/a$, where *a* and *b* are its semi-major and semi-minor axis. That means when $a=b$ and $\varepsilon =1$, the magnetization rotation rule should be given by $\alpha _{Halbach}\,\, =$ 2$\theta$. The other extreme, when ellipticity is zero, corresponds to a two parallel plane system (which of course is of no practical use) and has an optimum magnetization setting that all the individual magnets in both the planes should be aligned in the same direction. From a magnetization vs azimuth angle ($\theta$) plot (see Fig. 1a) for the above two extreme cases, it can be seen that the circular Halbach 2$\theta$ rotation rule gives a straight line (shown with a black dashed line) while the zero ellipticity case results in a Heaviside function (or step function), shown with a violet dashed line. This suggests that for all the other ellipticity values the curve should be somewhere in between the two extremes.

In the next two sections a few hypotheses are presented for finding the best rotation rule.

## Shape dependent design hypothesis (SDH)

It is shown in the previous section that the magnetization rotation rules change with ellipticity values and so it can be hypothesized that the rotation rules should be shape-dependent. The shape is defined by drawing a normal at any point to the surface of an ellipse (see Fig. 1b). The angle that this normal makes with the horizontal is given by $$\theta _{n} = tan^{-1}\begin{pmatrix}\frac{\varepsilon ^2y}{x}\end{pmatrix}$$. For an infinite length circular cross-section where $\theta = \theta _{n}$, the Halbach rotation rule is simply rewritten as twice the surface normal angle (${i.e.~} 2\theta _{n}$). However, for an elliptical cross-section with a non-unit ellipticity, $\theta _{n}$ $\ne$ $\theta$, see Fig. 1b. Therefore the SDH magnetization rule takes the form, $\alpha _{SDH} =2\theta _n=~$2$$tan^{-1}\begin{pmatrix}\frac{\varepsilon ^2y}{x}\end{pmatrix}$$, where (*x*, *y*) is a point coordinate on the surface of the ellipse where the magnetization angle $\alpha _{SDH}$ is measured. If magnetization vs azimuthal angle is plotted for an ellipse with $\varepsilon =0.5$ then it is represented by a sigmoidal curve, see the solid green curve in Fig. 1a.

**Figure 2:**
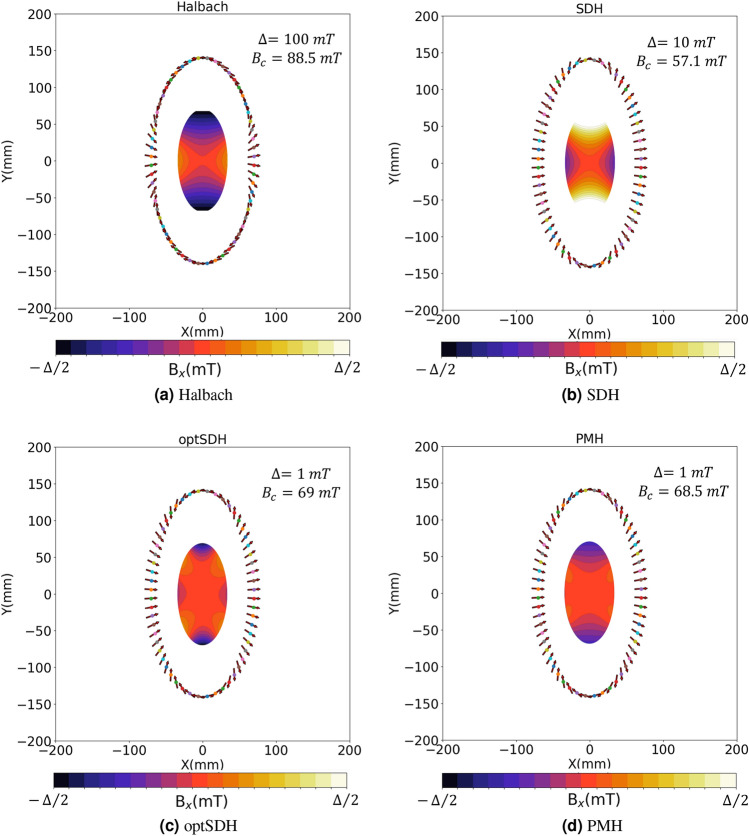
Magnetic field density map with magnetic flux lines for elliptical magnets with ellipticity, $\varepsilon = 0.5$ and elliptical ROI for (**a**) Halbach, (**b**) SDH, (**c**) optimized SDH and (**d**) PMH rotation rules. $\Delta$ and $B_c$ are the width and the central magnetic field values of the colorbar in the field density maps.

To check if using these new shape-dependent magnetization values gives better homogeneity than the Halbach configuration a magnetostatic simulations is performed of a large magnet array designed using the SDH. A large length-to-diameter ratio (9:1) is kept for these simulations to minimize the finite-length effects. The individual permanent magnets were simulated using the dipole approximation, as in many previous publications. Figure 2a,b show the field profiles when using Halbach’s 2$\theta$ and the SDH rotation rule, respectively. These profiles represent the dominant magnetic field component ($B_x$) at the central cross-section along the elliptical magnets. The results are tabulated in Table 1. The inhomogeneity values (defined as $\frac{B_{max}-B_{min}}{B_{mean}}\times 10^6$) are calculated inside an elliptic region of interest (ROI) with half the radial dimension and about 15$\%$ of the length of the simulated magnet. The results show that shape-dependent rotation rules give approximately a fourfold reduction in the inhomogeneity values as compared to the Halbach arrangement.

## Optimized shape dependent design hypothesis (optSDH)

he SDH rotation rule presented above gives better homogeneity than the Halbach rotation rule, but the natural question is if this is the most optimum way to orient the individual magnets or can another improved rotation rule delivering even better field homogeneity be derived? One way to optimize the rule shown in Fig. 1a is to add in a factor $\widetilde{m}$ which modifies the rotation rule to $\alpha _{optSDH}=~$2$$tan^{-1}\begin{pmatrix}\frac{\widetilde{m}\varepsilon ^2y}{x}\end{pmatrix}$$ which corresponds to an additional degree of freedom in defining a particular sigmoidal curve. Similar to the last section an example case of $\varepsilon = 0.5$ can be taken and a magnetostatic calculation for a long cylinder can be performed and the $\widetilde{m}$ value which delivers the best homogeneity could be found. As shown later in “Effect of ellipticity on homogeneity of the generated field”, it is found that optimized values of $\widetilde{m}$ for different ellipticities $\varepsilon$ is of the form $\frac{1}{m\varepsilon }$. Thus the optimized SDH rotation rule becomes $\alpha _{optSDH}=~$2$$tan^{-1}\begin{pmatrix}\frac{\varepsilon y}{mx}\end{pmatrix}$$. By performing this optimization one can get about 420 times improvement in the inhomogeneity values (for $m = 1.35$) as compared to the Halbach system as tabulated in Table 1. The corresponding 2D field profile is shown in Fig. 2c. The magnetization vs azimuth angle ($\theta$) plot for optSDH is shown in Fig. 1a with a solid blue curve, and as can be seen represents a different sigmoidal function than the green SDH curve.

This is a very promising outcome: however, one can recognize that the SDH or optSDH method has no direct interpretation connected with the physics of magnetic fields. In the next section, this will be addressed.

## Permanent magnet hypothesis (PMH)

In this section, a second hypothesis is formulated that is more intuitive with respect to the underlying physics. It will help us arrive at an optimized solution as shown in the previous section, but now directly from magnetostatic calculations. It is known^3^ that for an infinitely long cylindrical permanent magnet with a circular cross-section, magnetized perfectly in one direction, the magnetic field lines just outside the magnet have the form $$M=M_0\begin{pmatrix}sin\theta \\ cos\theta \end{pmatrix}$$. This means that the magnetic field vectors emanating from a circular permanent magnet rotate at a 2$\theta$ rate, which is the same as the Halbach rotation rule for an infinitely long circular Halbach array. That lays the foundation of the second hypothesis which is named the permanent-magnet hypothesis (PMH). The hypothesis suggests that the magnetization of individual magnets which form the hollow cylindrical array of a specific cross-section should align with the magnetic field vectors emanating from each point on the periphery of an infinitely long virtual permanent magnet (VPM) of the same cross-section geometry.

**Figure 3:**
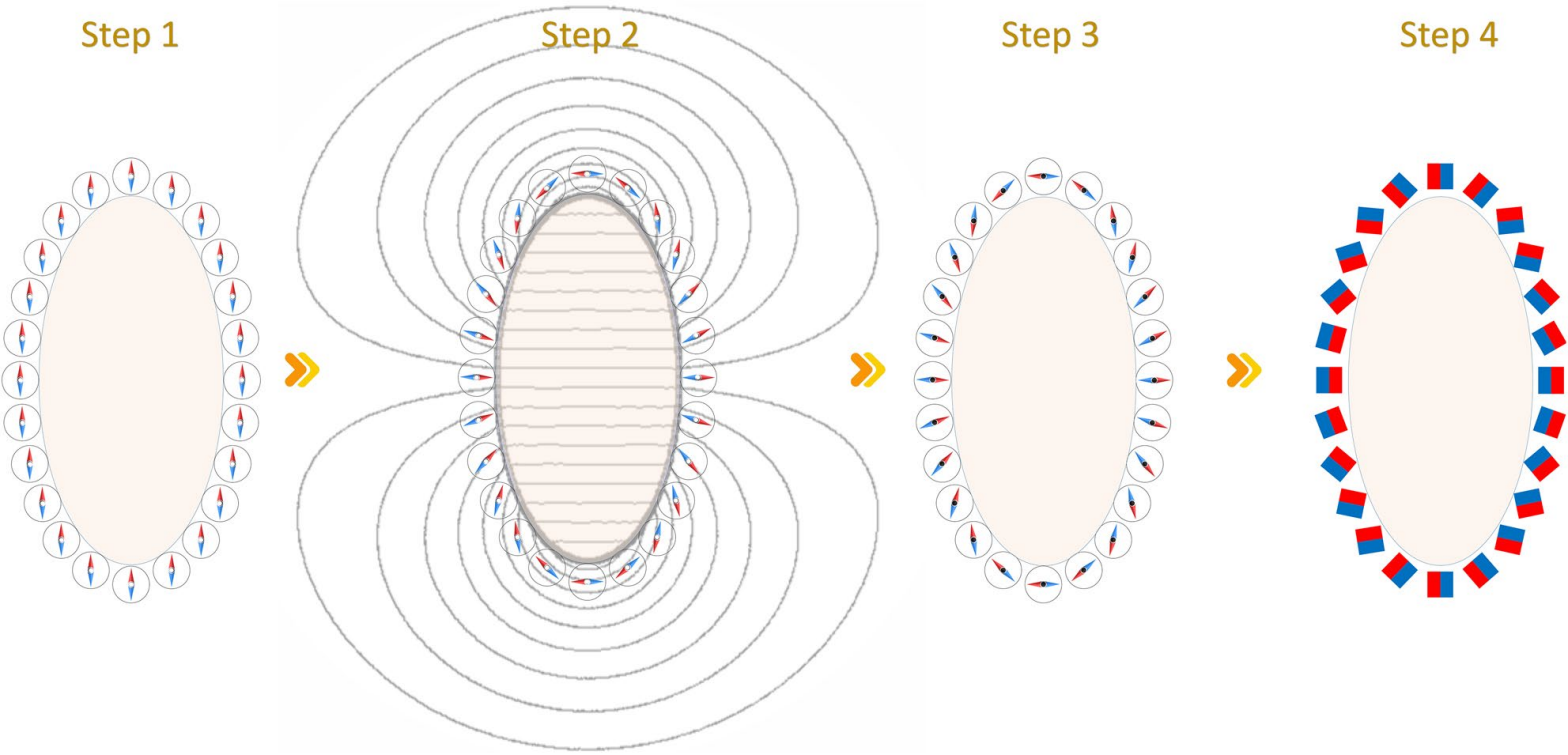
Steps to apply the PMH. In step 1, multiple freely suspended magnetic needles around a desired elliptical region of interest are hypothetically placed. In step 2, a virtual permanent magnet (VPM) of the same ellipticity is set in place and let the magnetic needles rearrange themselves under its influence. In step 3, the magnetic needles are pinned and the VPM is removed. The pinned position of the magnetic needles at step 3 shows the magnetization direction obtained using the PMH. Finally, in step 4, the magnetic needles are replaced with permanent magnets with their magnetization along the magnetic needles’ direction.

To understand how PMH would work, let us take the example of the elliptical magnet array with ellipticity $\varepsilon =0.5$. To create a magnet array that generates a uniform homogeneous dipole field along the x-axis, the steps to apply the PMH would be the following (see Fig. 3):

Step 1. Many hypothetical magnetic needles are placed in the periphery of the ellipse, all aligned along one direction, say along the y-axis. These magnetic needles are free to rotate and align with the local magnetic field.
Step 2. Then a virtual permanent magnet (VPM) of the same size as the ellipse, magnetized in the x-direction is placed in the center. Because of this VPM, each needle in the periphery will rotate and align towards the emanating field it experiences from the VPM. Practically, the magnetic field simulations for the VPM are performed using the Finite Element Method Magnetics (FEMM) open-source package (femm v4.2), which solves for a 2D planar field profile.
Step 3. Now the position of these needles are locked and the VPM is removed.
Step 4. The permanent magnets are then placed along the pinned needles which give the magnetization using the PMH.

In Fig. 1a for $\varepsilon =0.5$, the PMH magnetization solution is shown with red color square markers. Here it can be seen that the PMH magnetization rule comes very close to the optSDH solution presented in the last section. To compare the homogeneity of the field generated by the PMH magnetization rule a magnetostatic simulation of a long elliptical cylinder with $\varepsilon =0.5$ is performed. The corresponding 2D field profile is shown in Fig. 2d. As laid down in Table 1, the homogeneity of this magnet is much better than the Halbach and SDH rules and approaches the design obtained using optSDH method. The PMH is especially interesting because it is not an optimization technique like the optSDH but rather has its origin in the shape of the magnet and properties of the magnet and the environment. Thus PMH can easily be extended to arbitrarily shaped magnets and it is not restricted to circular or elliptical shaped cross-sections.

In the next section, it is shown how the homogeneity of the magnet array changes with ellipticity values for different rotation rules and also the difference in homogeneity observed between the PMH and optSDH is discussed.

**Table 1:** Comparison of different rotation rules for a long ($L/R\approx 9$) length elliptical magnet ($\varepsilon =0.5$).

	Halbach	SDH	optSDH	PMH
Bx (mT)	83	58	69	68
Homogeneity [ppm]	221,702	51071	528	1015

## Effect of ellipticity on homogeneity of the generated field

As mentioned earlier Küstler^11^ studied the effect of ellipticity on the homogeneity of the field, and found that the optimum ellipticity is around 1.174. This result was, however, obtained using Halbach rotation rules. The effect of ellipticity on mean magnetic field values for Halbach rotation rules has also been studied^29,34,35^. As it is shown above that the PMH and optSDH provide much better rotation rules for the same ellipticity value, so in this section the variation of homogeneity and mean magnetic field values for different ellipticity values when the magnetization is arranged using the PMH, SDH and optSDH approaches is investigated.

**Figure 4:**
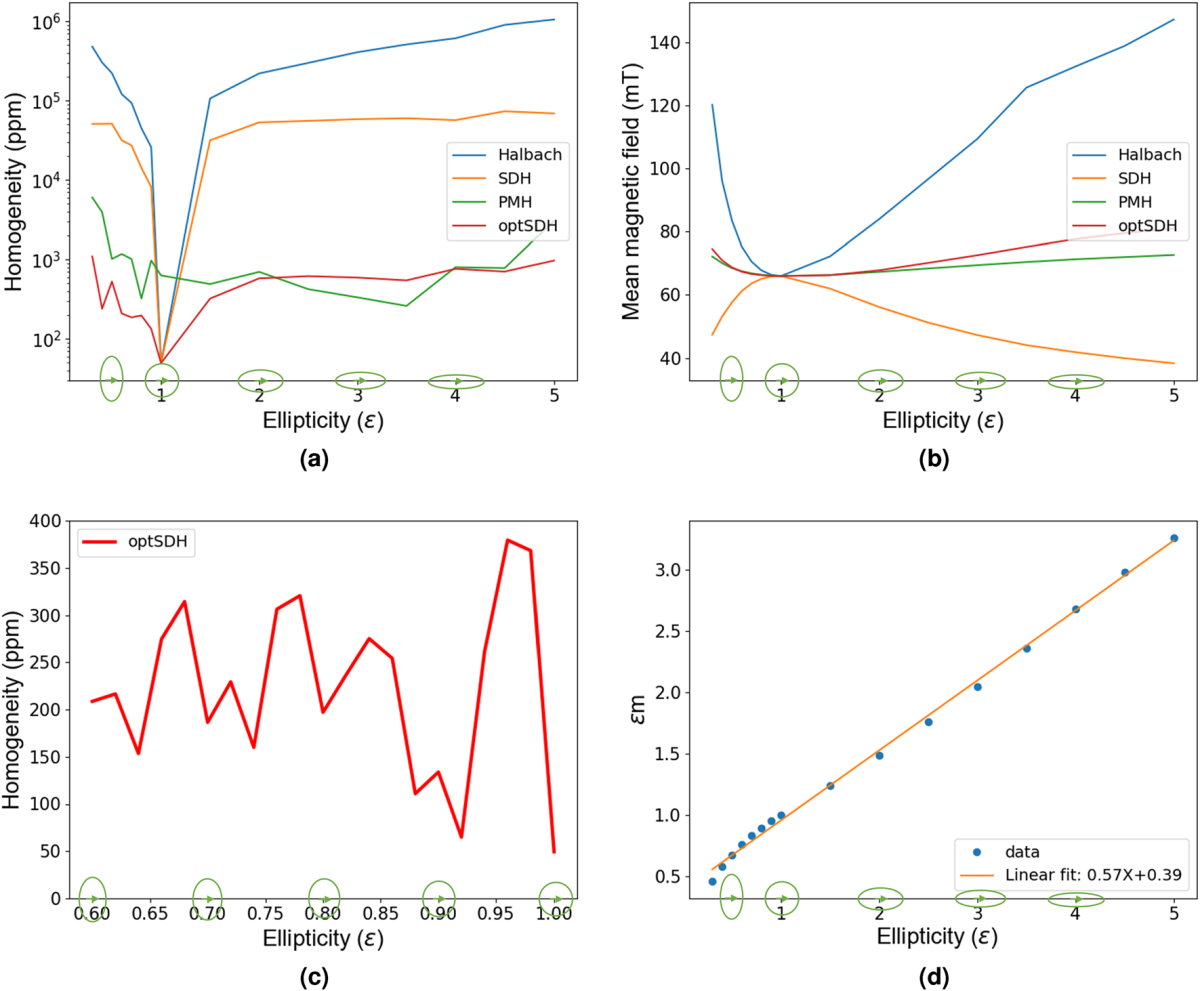
(**a**) Variation in homogeneity with ellipticity for the Halbach (blue curve), SDH (orange), optimized SDH (green) and PMH (red) rotation rules are shown in the same plot. The homogeneity of the field is measured in an elliptical ROI of the same ellipticity as the magnet. These values are plotted on a logarithmic scale. The green ellipses in the bottom show the different elliptic magnet designs corresponding to the ellipticity values along with the direction of the main magnetic field component. (**b**) Mean magnetic field as a function of ellipticity. Here it can be seen that if the target application demands the highest magnetic flux then Halbach rotation rules gives better results. (**c**) This represents a similar inhomogeneity variation plot as (**a**) for the optimized SDH rotation rule but taken at finer steps (0.02) along the ellipticity axis. (**d**) The inverse variation of $\widetilde{m}$ with eccentricity ($\varepsilon$) values, i.e., $\widetilde{m}=\frac{1}{m\varepsilon }$ is shown by plotting $m\varepsilon$ against $\varepsilon$. The *m* values here are optimized for different ellipticity values and are found to be on average around 0.57, as determined from the linear fit.

Similar to the approach of Küstler^11^, the amount of magnetized material is kept constant in the ellipticity analysis. In our case, this means keeping the number of permanent magnets the same, which in turn implies that all the ellipses with different ellipticity should have the same circumference as the magnets are arranged at equal distance from each other. Elliptical magnet arrays with ellipticity values ranging from 0.3 to 5 is studied here and the results are compared with the standard Halbach rotation rule. The ROI is defined also as an elliptic region with half the radial dimension (major and minor radii) and about 15$\%$ of the length of the whole simulated magnet, i.e. the ellipticity of the ROI is kept the same as that of the corresponding magnet. Figure 4a,b shows how the homogeneity and the mean magnetic flux strength of the field inside the magnet vary with ellipticity. For any non-unitary ellipticity values, it can be seen that the SDH, optSDH and PMH perform much better than Halbach rotation rules in terms of homogeneity of the field. However, at unit ellipticity, i.e., circular cross-section, the PMH inhomogeneity values are higher than for the Halbach. This is because the PMH magnetization values are sensitive to the accuracy of the FEMM calculations of the virtual permanent magnet, which depend on the size of the simulated magnetic domain, the meshing size, boundary conditions etc. These factors, of course, also affect the results for the non-unit ellipticity values. This is the reason why in Table 1 the field homogeneity for the PMH case was not as good as that for the optSDH. An exact analytical magnetostatic solution for the elliptical cross-section VPM would improve the calculations.

Next, the homogeneity values for finer ellipticity steps ranging from 0.6 to 1.0 with a step of 0.02 is calculated as shown in Fig. 4c for an optimized shape-dependent hypothesis (optSDH) rotation rule. What can be seen here is that the trend which looks rather smooth in Fig. 4a actually has multiple local minima and maxima. For sub-unit ellipticity values, the lowest inhomogeneity dip occurs around $\varepsilon =0.92$ with an inhomogeneity value of around 64 ppm at 66 mT mean-field with an optimized $m=1.045$. For comparison, the Halbach rotation rule for a similar elliptical magnet of $\varepsilon =0.92$ gives an inhomogeneity of 20562 ppm at with a mean-field of 66 mT. In Fig. 4d it is also shown how the optimized $\widetilde{m}$ values obtained for the optSDH case change with the ellipticity. This gives the rational behind the $\frac{1}{m\varepsilon }$ trend of $\widetilde{m}$ and the $\alpha _{optSDH}$ magnetization rule presented earlier.

## Finite-size elliptical magnet arrays

**Figure 5:**
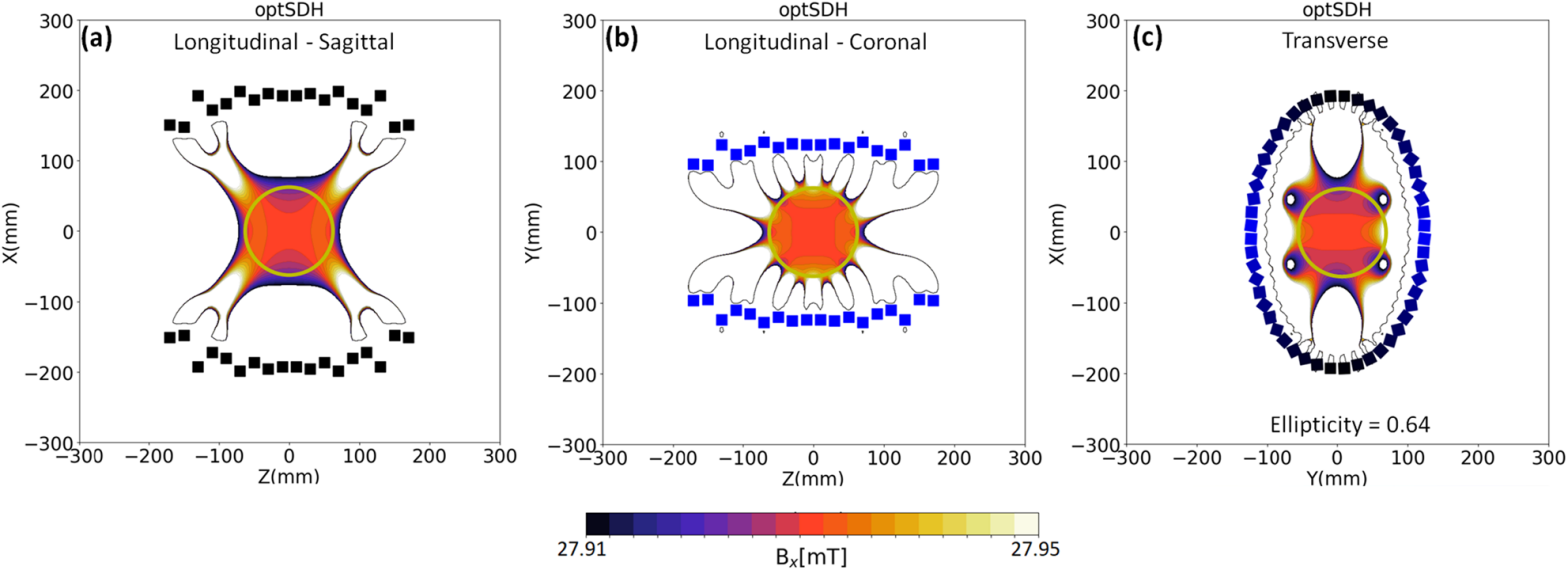
Design of an elliptical magnet ($\varepsilon =0.64$) with a length equal to its diameter, and variable diameter rings: 2D contour plots showing the magnetic field profile in the (**a**) sagittal, (**b**) coronal and (**c**) transverse directions. The colour bar is the same for the three field contour plots. The magnets are coloured from black to blue from the semi-major to the semi-minor axis. A spherical ROI is depicted by the solid circle in the three plots.

In the previous section the optimum magnetization directions are calculated for a long ($L/R\approx 9$) magnet array with an elliptical cross-section. Similar to Halbach circular arrays, when the length-to-diameter ratio is reduced, the homogeneity is reduced. Various methodologies derived for the circular cross-section magnet can be used and applied for this case. Here the result for an elliptical magnet array with a length-to-diameter ratio of 1:1 is presented, and then the design is optimized by varying the size of different elliptical rings (keeping the ellipticity the same) using a genetic algorithm. For this, a custom-built code is prepared using an evolutionary computation framework called Distributed Evolutionary Algorithms in Python or DEAP. Figure 5 shows the magnetic field profiles for the two longitudinal and one transverse plane at the centre of the magnet. The spherical region of interest of diameter 125 mm is defined at the centre of the magnet as shown by the solid circle in the three planes. The field inside the ROI has a mean-field strength of 28 mT and homogeneity of 672 ppm. The details of the final magnet design are provided in Table 2. The colour of the magnets in Fig. 5 changes from black to blue on going from the semi-major to the semi-minor axis.

**Figure 6:**
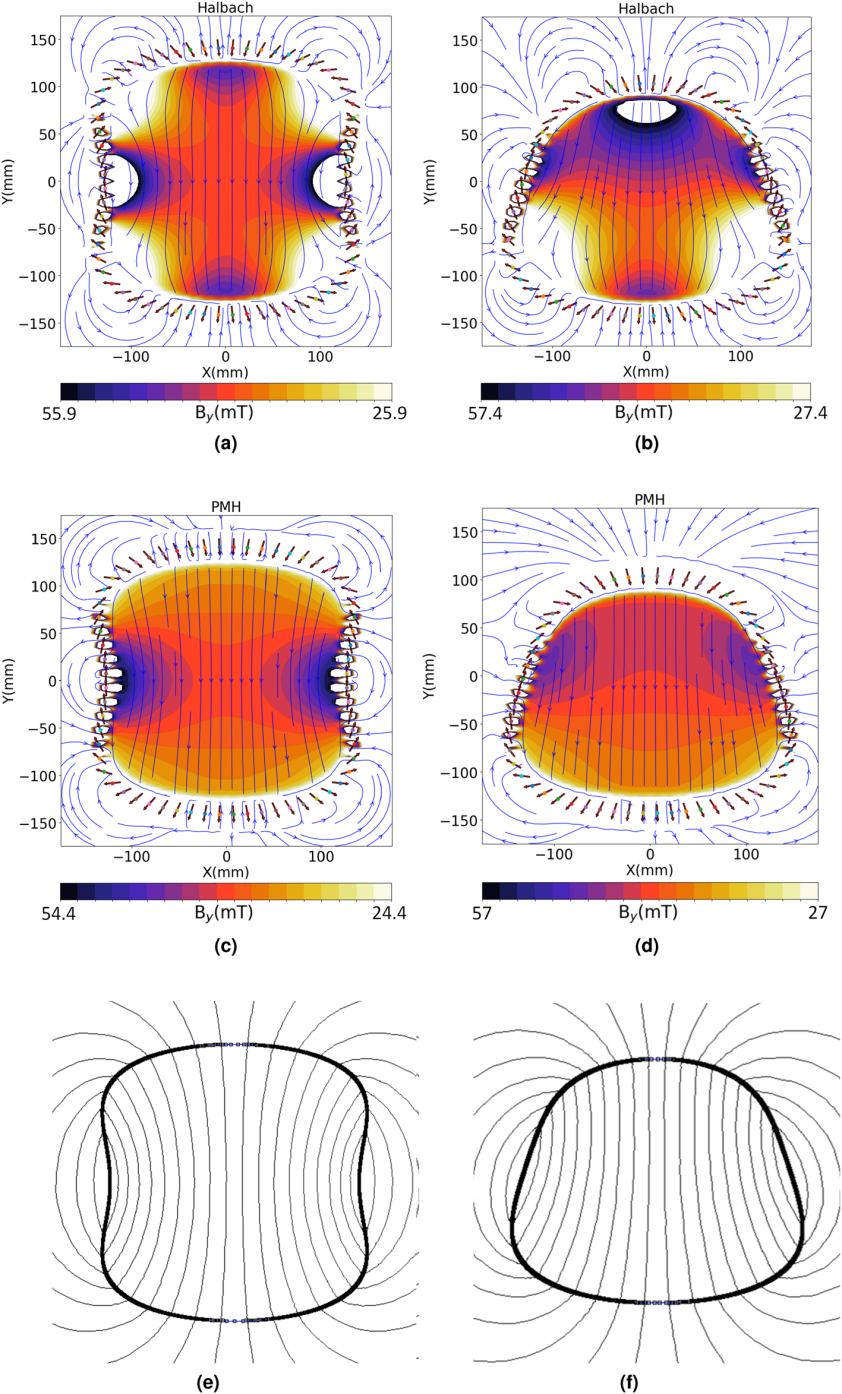
Magnetic field profiles when using (**a**) Halbach’s 2$\theta$, (**c**) PMH rotation rule and the (**e**) the corresponding VPM for the arbitrarily shaped magnet of a non-circular cross-section of the following form: $ax^2-by^2+cy^4=d$. This magnet has symmetry in the x- and y- directions. A second example magnet is also simulated with the following form: $ax^2-by+cy^4=d$, where the y-symmetry is lost. The field profiles are presented for (**b**) Halbach and (**d**) PMH rotation rules. The corresponding VPM is shown in (**f**). These profiles are for the dominant magnetic field component ($B_y$) at the central cross-section of long magnets (length $\approx$ 1 m) to avoid end effects). The magnetic field lines are also juxtaposed to show the difference between PMH and Halbach situations for the two magnets. The thick arrows mark the position and orientation of the permanents magnets forming the entire magnet array.

**Table 2:** Variable-diameter elliptical magnet ($\varepsilon =0.64$)—design parameters.

	R0	R1	R2	R3	R4	R5	R6	R7	R8
Z [mm]	10	30	50	70	90	110	130	150	170
R [mm]	193	196	187	199	181	172	193	148	151
Number of magnets	52	56	52	56	48	48	52	40	40

## Arbitrary cross-section shaped magnetic array systems

The intuitive PMH approach is not limited to circular or elliptic cross-sections, but can include cross-sections with any arbitrary shape. Two cross-sections are shown here for which the magnetization rule is extracted by using a VPM (Fig. 6e,f), magnetized in the desired direction. Using these magnetization rules long magnet arrays (Length $\approx$ 1m) of these arbitrary cross-sections is constructed and magnetostatic calculations are performed. One example has two axes of symmetry, and the other a single symmetrical axis. The magnetic field results thus obtained for the symmetric and non-symmetric magnet design are shown in Fig. 6a–d respectively. Here the magnetic field profiles are shown for Halbach and PMH configurations. The colour bar used in all the figures has the same window range of 30 mT centred around the mean $B_y$ field at the middle of the magnet. What can be seen is that the PMH (c,d) results in a much higher uniformity in the magnetic field as compared to applying Halbach (a,b) rotation rule to these magnets. The quantitative homogeneity improvements are listed in Table 3 and are calculated for a spherical ROI. Although the field is more homogeneous with the PMH rotation rule, the homogeneity values are not as good as that for circular or elliptical cross-sections. However, for an arbitrarily shaped magnet, the PMH could serve as a good starting point to find the optimized magnetization rule.

Important to also note is that the field lines inside the hollow magnet array using the PMH rule follow closely the field lines inside the corresponding VPMs shown in Fig. 6e,f. This suggests that improving the magnetic homogeneity inside an arbitrarily shaped magnet array could be achieved by improving the uniformity of the magnetic field inside the corresponding VPM. Additionally, it is also possible according to the application in-hand, that an iron yoke might be used for concentrating and channelling the magnetic field lines in a desired manner. In that case, the iron yoke also has to be included into the simulations.

**Table 3:** Arbitrary shaped magnets: Halbach and PMH comparison.

	sym-Halbach	sym-PMH	nonsym-Halbach	nonsym-PMH
Bx (mT)	40.9	39.4	42.4	42
Homogeneity [ppm]	186,884	155,350	372,985	103,425

## Conclusion

In this paper both numerically-optimized and more intuitive-based approaches to designing non-circular Halbach arrays are presented. Special focus is placed on designing magnets with elliptical cross-sections and it is shown that the usual Halbach rotation rule can be significantly improved in terms of deriving optimal homogeneity. For the particular geometries studied, the results presented here show an almost 420 times reduction in the inhomogeneity of the magnetic field inside a magnet with an ellipticity of 0.5 using an optimized shape-dependent hypothesis. A permanent magnet hypothesis (PMH) is then introduced which arrives at a similar rotation rule but without requiring any numerical optimization. The central theme of the PMH is that the magnetization orientations of the magnets forming the Halbach array can be deduced from the magnetic field lines originating from a virtual permanent magnet filling the bore of the Halbach array. These rotation rules are also compared for multiple ellipticity values for the cross-section. Then a design of an elliptical cross-section finite-length variable diameter magnet is shown using the optimized magnetization rotation rule (Supplementary Information S1).

## Supplementary Information

Supplementary Information.

## Data Availability

The database containing the data generated and/or analysed during this study performed related to the permanent magnet hypothesis (PMH) is provided as a compressed zip folder in the supplementary material.
